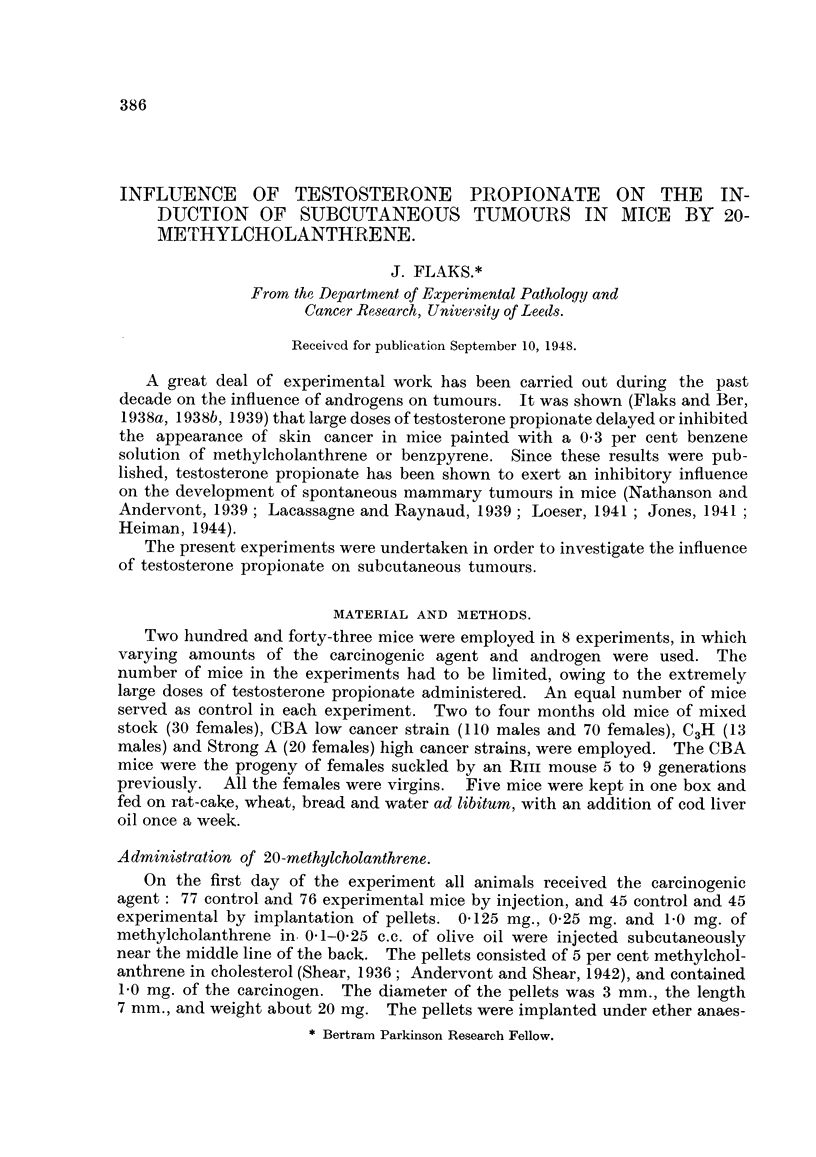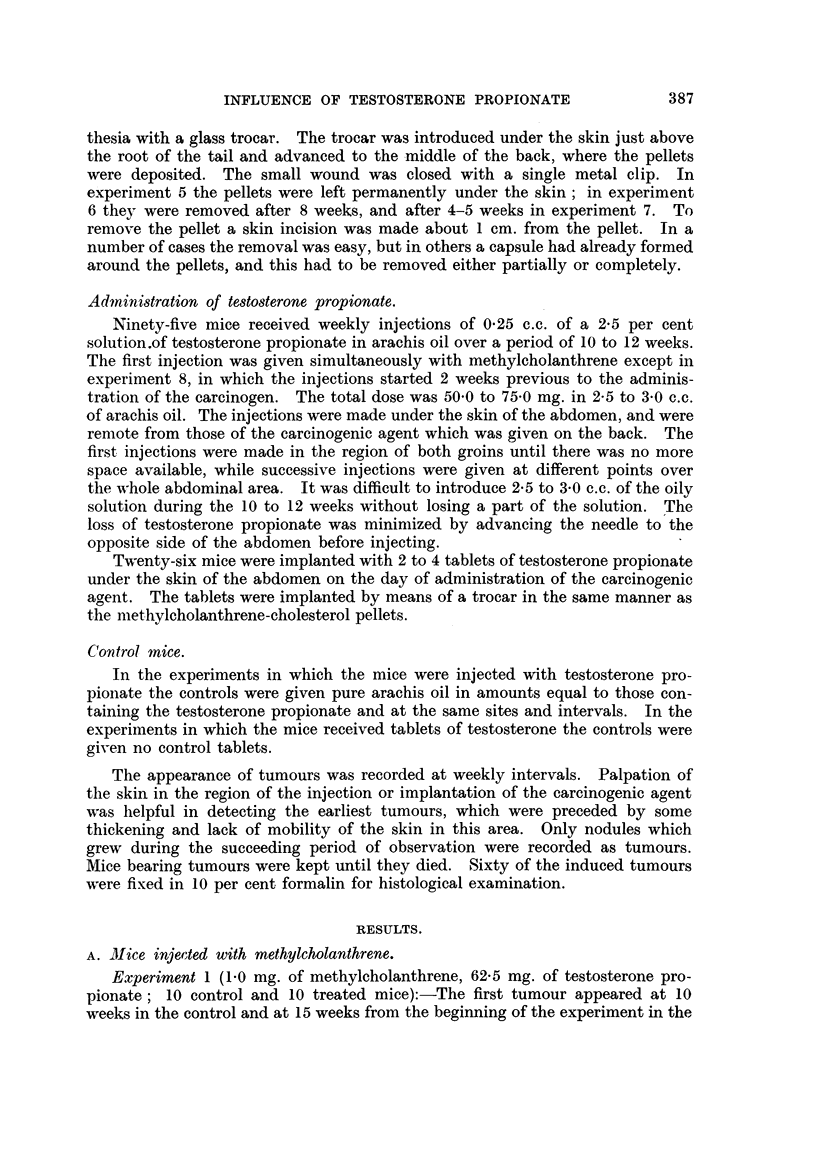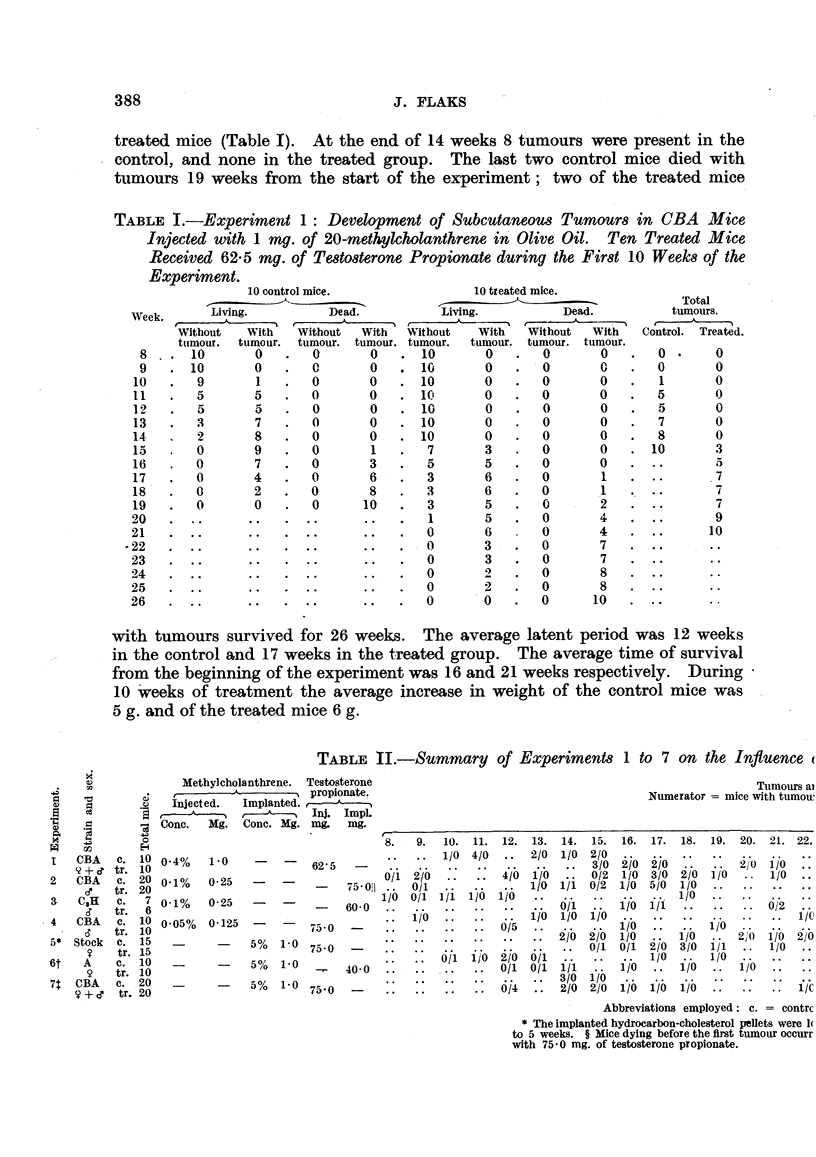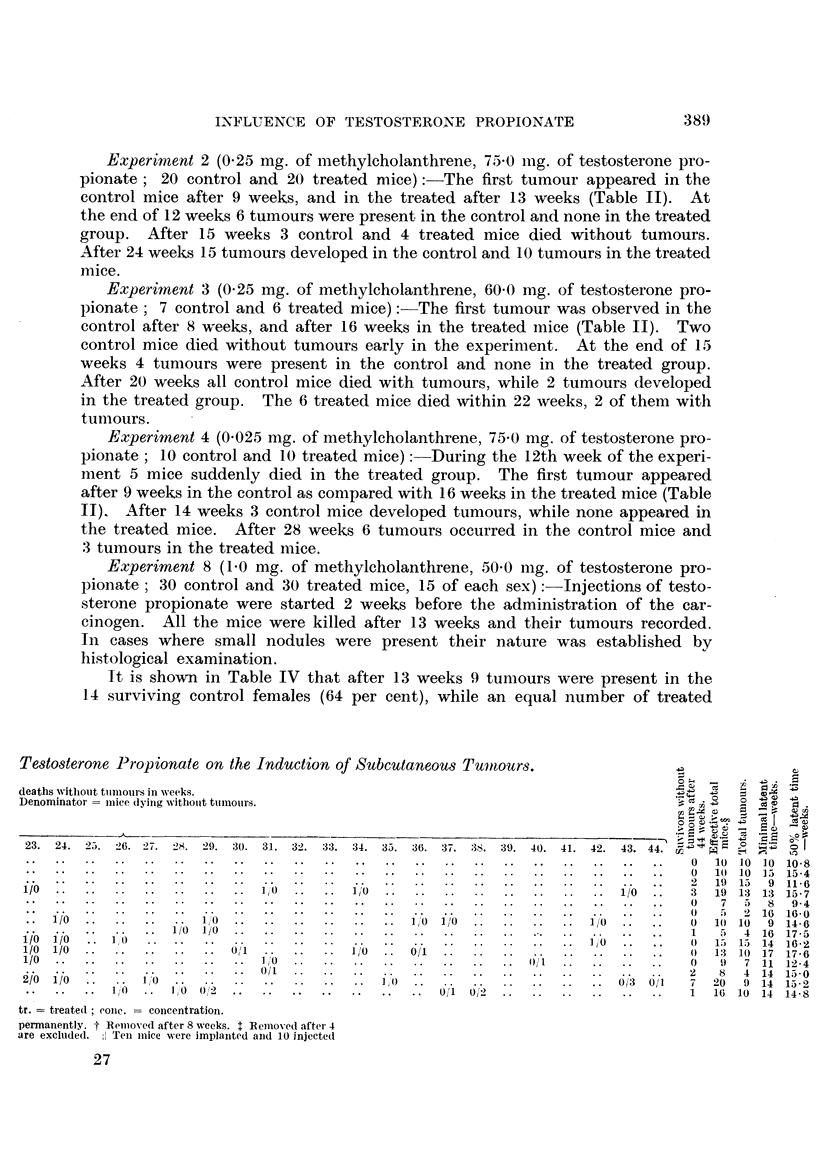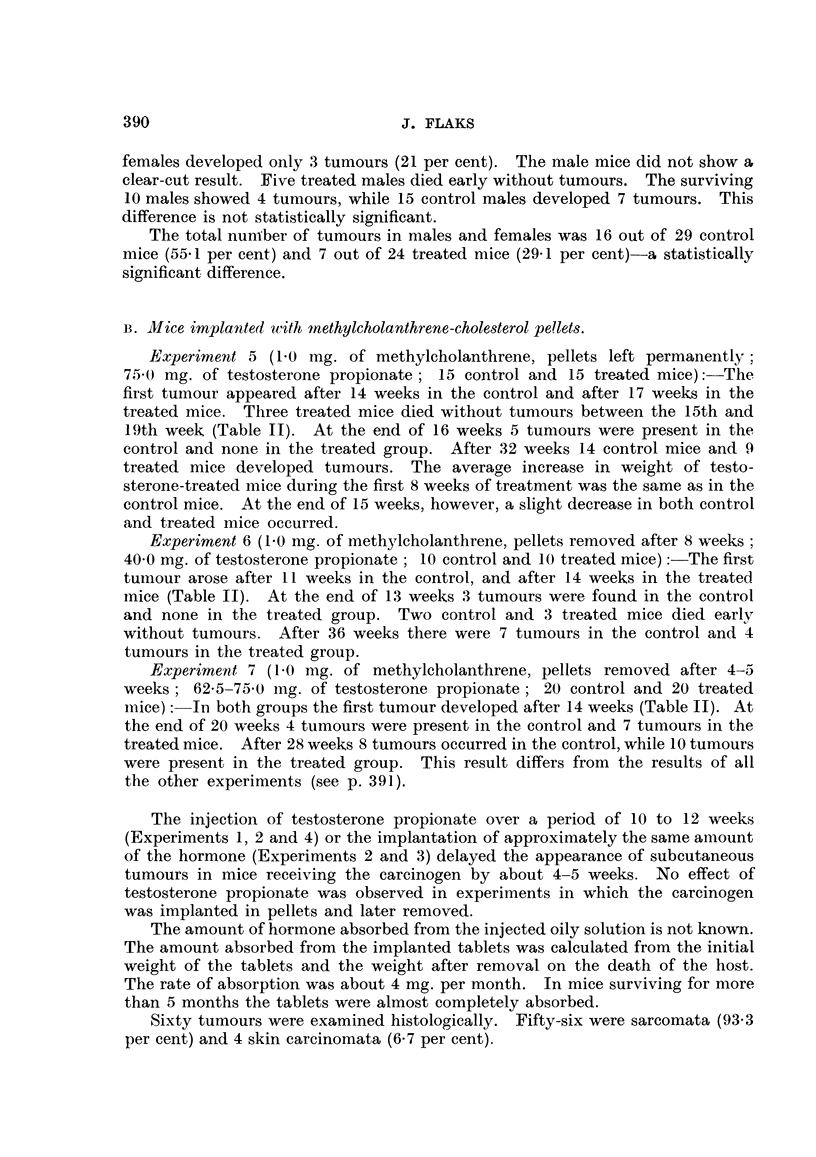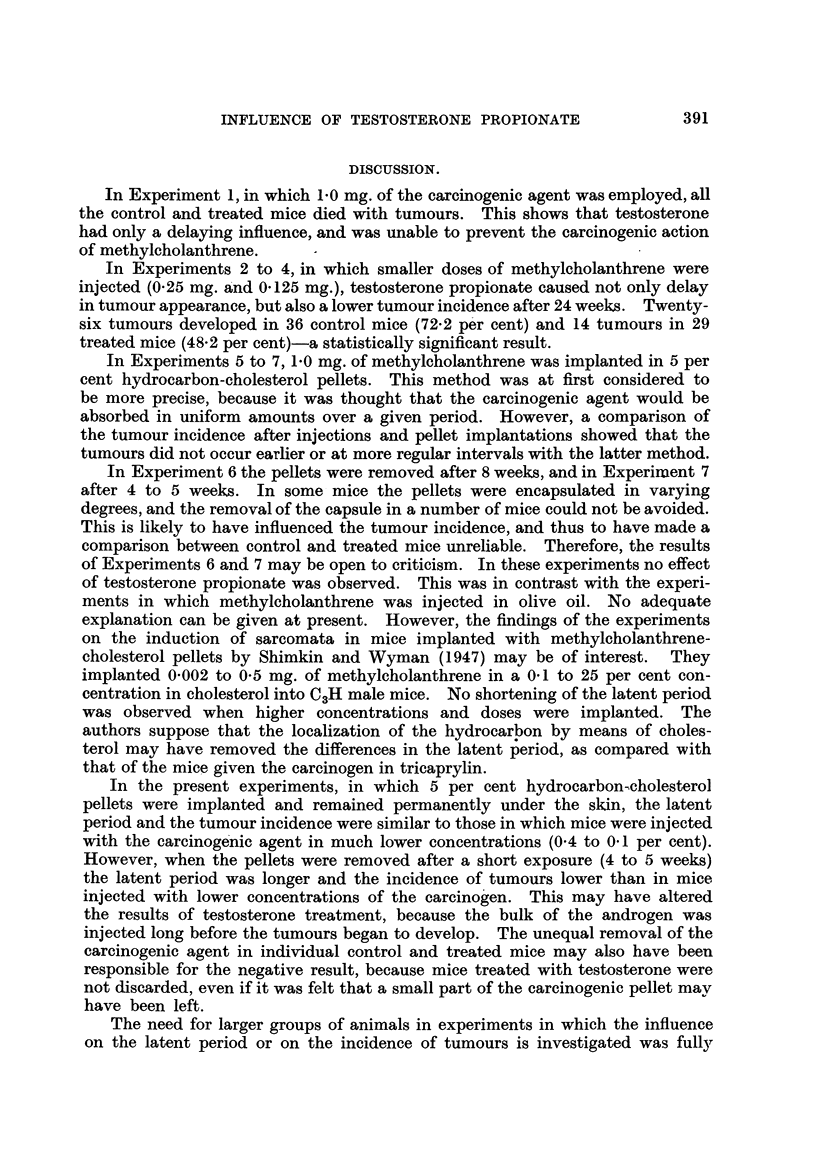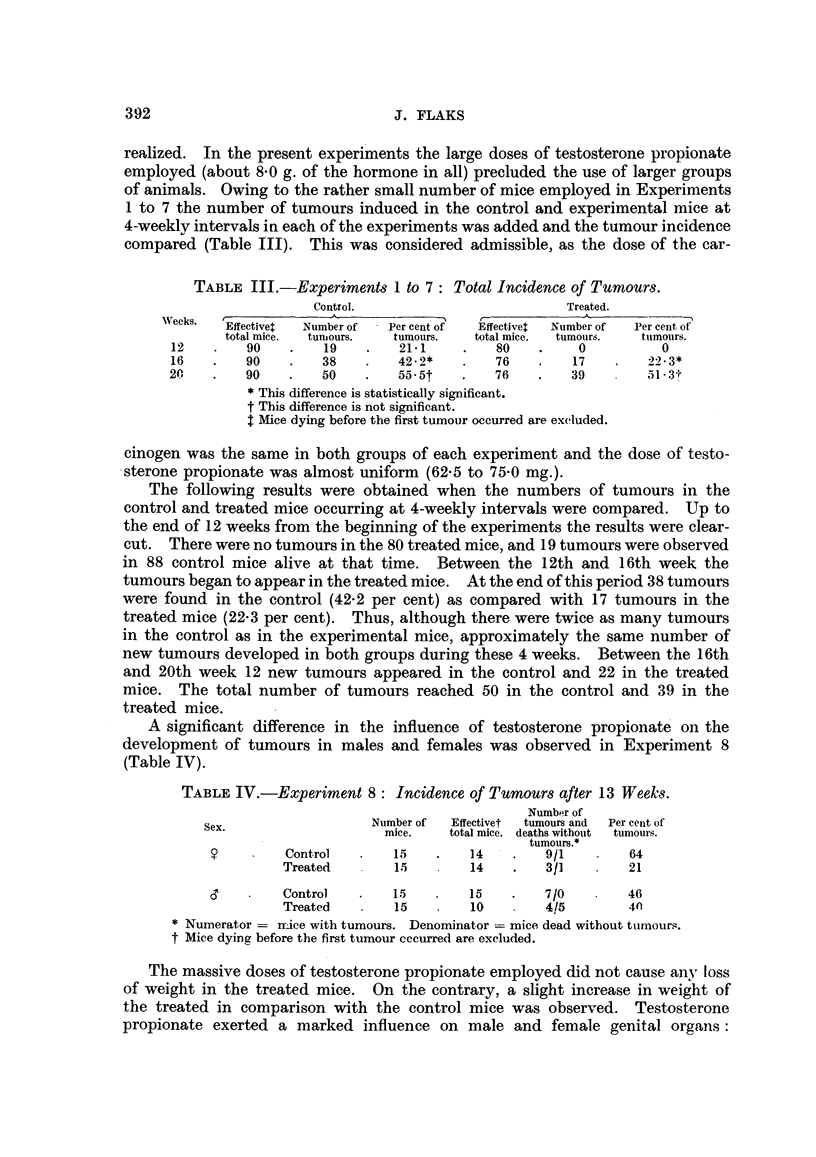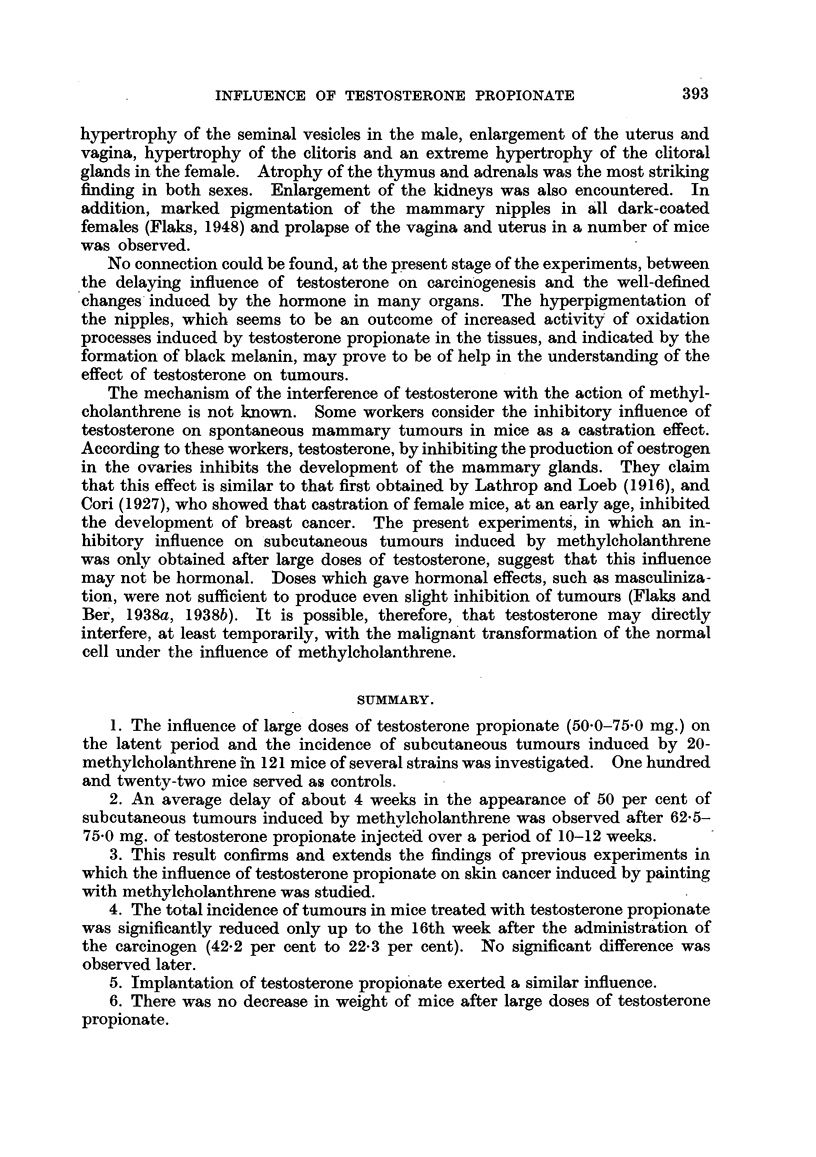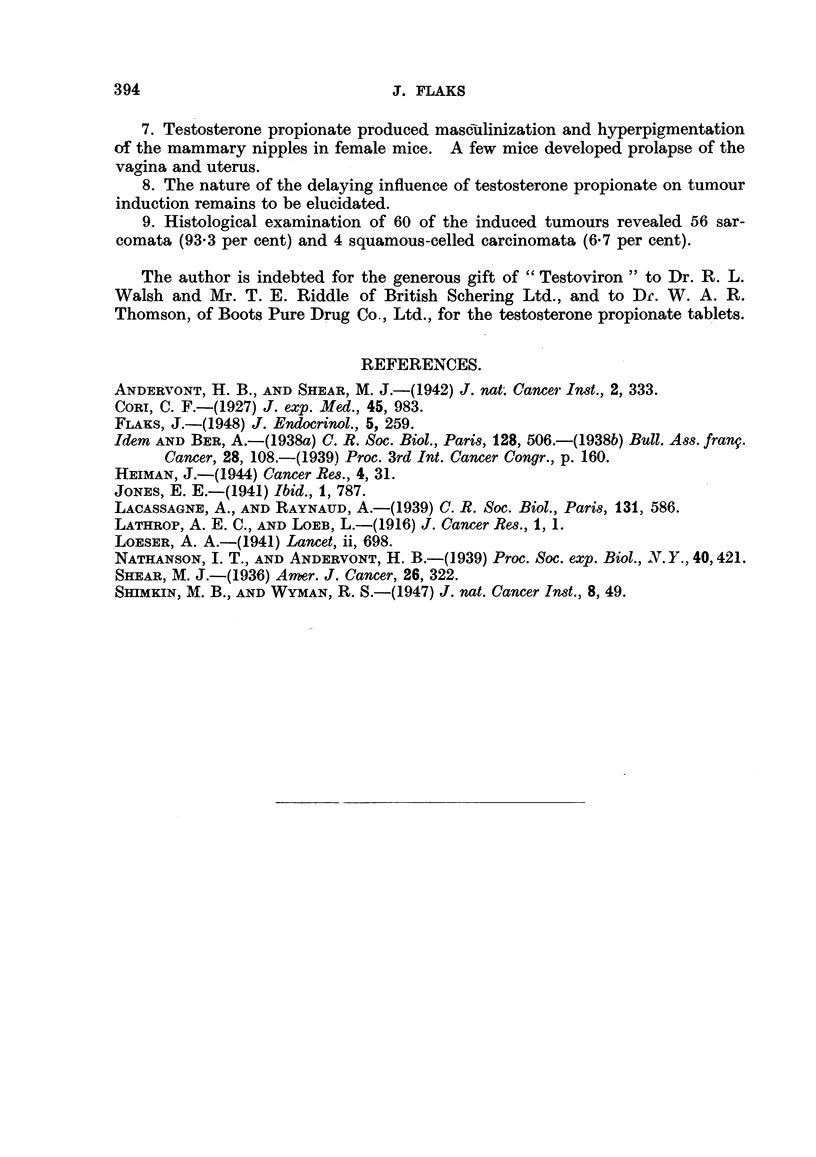# Influence of Testosterone Propionate on the Induction of Subcutaneous Tumours in Mice by 20-Methylcholanthrene

**DOI:** 10.1038/bjc.1948.43

**Published:** 1948-12

**Authors:** J. Flaks


### INFLUENCE OF TESTOSTERONE PROPIONATE ON THE INDUCTION OF SUBCUTANEOUS TUMOURS IN MICE BY 20-METHYLCHOLANTHRENE.

J. FLAKS.*

*From the Department of Experimental Pathology and Cancer Research, University of Leeds.*

Received for publication September 10, 1948.

A great deal of experimental work has been carried out during the past decade on the influence of androgens on tumours. It was shown (Flaks and Ber, 1938*a*, 1938*b*, 1939) that large doses of testosterone propionate delayed or inhibited the appearance of skin cancer in mice painted with a 0·3 per cent benzene solution of methylcholanthrene or benzpyrene. Since these results were published, testosterone propionate has been shown to exert an inhibitory influence on the development of spontaneous mammary tumours in mice (Nathanson and Andervont, 1939 ; Lacassagne and Raynaud, 1939 ; Loeser, 1941 ; Jones, 1941 ; Heiman, 1944).

The present experiments were undertaken in order to investigate the influence of testosterone propionate on subcutaneous tumours.

### MATERIAL AND METHODS.

Two hundred and forty-three mice were employed in 8 experiments, in which varying amounts of the carcinogenic agent and androgen were used. The number of mice in the experiments had to be limited, owing to the extremely large doses of testosterone propionate administered. An equal number of mice served as control in each experiment. Two to four months old mice of mixed stock (30 females), CBA low cancer strain (110 males and 70 females), $C_3H$ (13 males) and Strong A (20 females) high cancer strains, were employed. The CBA mice were the progeny of females suckled by an RIII mouse 5 to 9 generations previously. All the females were virgins. Five mice were kept in one box and fed on rat-cake, wheat, bread and water *ad libitum*, with an addition of cod liver oil once a week.

### *Administration of* 20-*methylcholanthrene.*

On the first day of the experiment all animals received the carcinogenic agent : 77 control and 76 experimental mice by injection, and 45 control and 45 experimental by implantation of pellets. 0·125 mg., 0·25 mg. and 1·0 mg. of methylcholanthrene in 0·1–0·25 c.c. of olive oil were injected subcutaneously near the middle line of the back. The pellets consisted of 5 per cent methylcholanthrene in cholesterol (Shear, 1936 ; Andervont and Shear, 1942), and contained 1·0 mg. of the carcinogen. The diameter of the pellets was 3 mm., the length 7 mm., and weight about 20 mg. The pellets were implanted under ether anaes-

* Bertram Parkinson Research Fellow.

thesia with a glass trocar. The trocar was introduced under the skin just above the root of the tail and advanced to the middle of the back, where the pellets were deposited. The small wound was closed with a single metal clip. In experiment 5 the pellets were left permanently under the skin ; in experiment 6 they were removed after 8 weeks, and after 4–5 weeks in experiment 7. To remove the pellet a skin incision was made about 1 cm. from the pellet. In a number of cases the removal was easy, but in others a capsule had already formed around the pellets, and this had to be removed either partially or completely.

*Administration of testosterone propionate.*

Ninety-five mice received weekly injections of 0·25 c.c. of a 2·5 per cent solution of testosterone propionate in arachis oil over a period of 10 to 12 weeks. The first injection was given simultaneously with methylcholanthrene except in experiment 8, in which the injections started 2 weeks previous to the administration of the carcinogen. The total dose was 50·0 to 75·0 mg. in 2·5 to 3·0 c.c. of arachis oil. The injections were made under the skin of the abdomen, and were remote from those of the carcinogenic agent which was given on the back. The first injections were made in the region of both groins until there was no more space available, while successive injections were given at different points over the whole abdominal area. It was difficult to introduce 2·5 to 3·0 c.c. of the oily solution during the 10 to 12 weeks without losing a part of the solution. The loss of testosterone propionate was minimized by advancing the needle to the opposite side of the abdomen before injecting.

Twenty-six mice were implanted with 2 to 4 tablets of testosterone propionate under the skin of the abdomen on the day of administration of the carcinogenic agent. The tablets were implanted by means of a trocar in the same manner as the methylcholanthrene-cholesterol pellets.

*Control mice.*

In the experiments in which the mice were injected with testosterone propionate the controls were given pure arachis oil in amounts equal to those containing the testosterone propionate and at the same sites and intervals. In the experiments in which the mice received tablets of testosterone the controls were given no control tablets.

The appearance of tumours was recorded at weekly intervals. Palpation of the skin in the region of the injection or implantation of the carcinogenic agent was helpful in detecting the earliest tumours, which were preceded by some thickening and lack of mobility of the skin in this area. Only nodules which grew during the succeeding period of observation were recorded as tumours. Mice bearing tumours were kept until they died. Sixty of the induced tumours were fixed in 10 per cent formalin for histological examination.

### RESULTS.

A. *Mice injected with methylcholanthrene.*

*Experiment* 1 (1·0 mg. of methylcholanthrene, 62·5 mg. of testosterone propionate ; 10 control and 10 treated mice):—The first tumour appeared at 10 weeks in the control and at 15 weeks from the beginning of the experiment in the treated mice (Table I). At the end of 14 weeks 8 tumours were present in the control, and none in the treated group. The last two control mice died with tumours 19 weeks from the start of the experiment; two of the treated mice

TABLE I.—*Experiment* 1 : *Development of Subcutaneous Tumours in CBA Mice Injected with* 1 *mg. of* 20-*methylcholanthrene in Olive Oil. Ten Treated Mice Received* 62·5 *mg. of Testosterone Propionate during the First* 10 *Weeks of the Experiment.*

| Week. | 10 control mice. Living. Without tumour. | With tumour. | Dead. Without tumour. | With tumour. | 10 treated mice. Living. Without tumour. | With tumour. | Dead. Without tumour. | With tumour. | Total tumours. Control. | Treated. |
|---|---|---|---|---|---|---|---|---|---|---|
| 8 | 10 | 0 | 0 | 0 | 10 | 0 | 0 | 0 | 0 | 0 |
| 9 | 10 | 0 | 0 | 0 | 10 | 0 | 0 | 0 | 0 | 0 |
| 10 | 9 | 1 | 0 | 0 | 10 | 0 | 0 | 0 | 1 | 0 |
| 11 | 5 | 5 | 0 | 0 | 10 | 0 | 0 | 0 | 5 | 0 |
| 12 | 5 | 5 | 0 | 0 | 10 | 0 | 0 | 0 | 5 | 0 |
| 13 | 3 | 7 | 0 | 0 | 10 | 0 | 0 | 0 | 7 | 0 |
| 14 | 2 | 8 | 0 | 0 | 10 | 0 | 0 | 0 | 8 | 0 |
| 15 | 0 | 9 | 0 | 1 | 7 | 3 | 0 | 0 | 10 | 3 |
| 16 | 0 | 7 | 0 | 3 | 5 | 5 | 0 | 0 | .. | 5 |
| 17 | 0 | 4 | 0 | 6 | 3 | 6 | 0 | 1 | .. | 7 |
| 18 | 0 | 2 | 0 | 8 | 3 | 6 | 0 | 1 | .. | 7 |
| 19 | 0 | 0 | 0 | 10 | 3 | 5 | 0 | 2 | .. | 7 |
| 20 | .. | .. | .. | .. | 1 | 5 | 0 | 4 | .. | 9 |
| 21 | .. | .. | .. | .. | 0 | 6 | 0 | 4 | .. | 10 |
| 22 | .. | .. | .. | .. | 0 | 3 | 0 | 7 | .. | .. |
| 23 | .. | .. | .. | .. | 0 | 3 | 0 | 7 | .. | .. |
| 24 | .. | .. | .. | .. | 0 | 2 | 0 | 8 | .. | .. |
| 25 | .. | .. | .. | .. | 0 | 2 | 0 | 8 | .. | .. |
| 26 | .. | .. | .. | .. | 0 | 0 | 0 | 10 | .. | .. |

with tumours survived for 26 weeks. The average latent period was 12 weeks in the control and 17 weeks in the treated group. The average time of survival from the beginning of the experiment was 16 and 21 weeks respectively. During 10 weeks of treatment the average increase in weight of the control mice was 5 g. and of the treated mice 6 g.

TABLE II.—*Summary of Experiments* 1 *to* 7 *on the Influence* (

| Experiment. | Strain and sex. | | Total mice. | Methylcholanthrene. Injected. Conc. | Mg. | Implanted. Conc. | Mg. | Testosterone propionate. Inj. mg. | Impl. mg. | Tumours ar Numerator = mice with tumou 8. | 9. | 10. | 11. | 12. | 13. | 14. | 15. | 16. | 17. | 18. | 19. | 20. | 21. | 22. |
|---|---|---|---|---|---|---|---|---|---|---|---|---|---|---|---|---|---|---|---|---|---|---|---|---|
| 1 | CBA | c. | 10 | 0·4% | 1·0 | — | — | 62·5 | — | .. | .. | 1/0 | 4/0 | .. | 2/0 | 1/0 | 2/0 | .. | .. | .. | .. | .. | .. | .. |
| | ♀ + ♂ | tr. | 10 | | | | | | | .. | .. | .. | .. | .. | .. | .. | 3/0 | 2/0 | 2/0 | .. | .. | 2/0 | 1/0 | .. |
| 2 | CBA | c. | 20 | 0·1% | 0·25 | — | — | — | 75·0‖ | 0/1 | 2/0 | .. | .. | 4/0 | 1/0 | .. | 0/2 | 1/0 | 3/0 | 2/0 | 1/0 | .. | 1/0 | .. |
| | ♂ | tr. | 20 | | | | | | | .. | 0/1 | .. | .. | .. | 1/0 | 1/1 | 0/2 | 1/0 | 5/0 | 1/0 | .. | .. | .. | .. |
| 3 | $C_3H$ | c. | 7 | 0·1% | 0·25 | — | — | — | 60·0 | 1/0 | 0/1 | 1/1 | 1/0 | 1/0 | .. | .. | .. | .. | .. | 1/0 | .. | .. | .. | .. |
| | ♂ | tr. | 6 | | | | | | | .. | .. | .. | .. | .. | .. | 0/1 | .. | 1/0 | 1/1 | .. | .. | .. | 0/2 | .. |
| 4 | CBA | c. | 10 | 0·05% | 0·125 | — | — | 75·0 | — | .. | 1/0 | .. | .. | .. | 1/0 | 1/0 | 1/0 | .. | .. | .. | .. | .. | .. | 1/[illegible] |
| | ♂ | tr. | 10 | | | | | | | .. | .. | .. | .. | 0/5 | .. | .. | .. | 1/0 | .. | .. | 1/0 | .. | .. | .. |
| 5* | Stock | c. | 15 | — | — | 5% | 1·0 | 75·0 | — | .. | .. | .. | .. | .. | .. | 2/0 | 2/0 | 1/0 | .. | 1/0 | .. | 2/0 | 1/0 | 2/0 |
| | ♀ | tr. | 15 | | | | | | | .. | .. | .. | .. | .. | .. | .. | 0/1 | 0/1 | 2/0 | 3/0 | 1/1 | .. | 1/0 | .. |
| 6† | A | c. | 10 | — | — | 5% | 1·0 | — | 40·0 | .. | .. | 0/1 | 1/0 | 2/0 | 0/1 | .. | .. | .. | 1/0 | .. | 1/0 | .. | .. | .. |
| | ♀ | tr. | 10 | | | | | | | .. | .. | .. | .. | 0/1 | 0/1 | 1/1 | .. | 1/0 | .. | 1/0 | .. | 1/0 | .. | .. |
| 7‡ | CBA | c. | 20 | — | — | 5% | 1·0 | 75·0 | — | .. | .. | .. | .. | .. | .. | 3/0 | 1/0 | .. | .. | .. | .. | .. | .. | .. |
| | ♀ + ♂ | tr. | 20 | | | | | | | .. | .. | .. | .. | 0/4 | .. | 2/0 | 2/0 | 1/0 | 1/0 | 1/0 | .. | .. | .. | 1/[illegible] |

Abbreviations employed: c. = contro

* The implanted hydrocarbon-cholesterol pellets were lo to 5 weeks. § Mice dying before the first tumour occurr with 75·0 mg. of testosterone propionate.

*Experiment* 2 (0·25 mg. of methylcholanthrene, 75·0 mg. of testosterone propionate ; 20 control and 20 treated mice) :—The first tumour appeared in the control mice after 9 weeks, and in the treated after 13 weeks (Table II). At the end of 12 weeks 6 tumours were present in the control and none in the treated group. After 15 weeks 3 control and 4 treated mice died without tumours. After 24 weeks 15 tumours developed in the control and 10 tumours in the treated mice.

*Experiment* 3 (0·25 mg. of methylcholanthrene, 60·0 mg. of testosterone propionate ; 7 control and 6 treated mice) :—The first tumour was observed in the control after 8 weeks, and after 16 weeks in the treated mice (Table II). Two control mice died without tumours early in the experiment. At the end of 15 weeks 4 tumours were present in the control and none in the treated group. After 20 weeks all control mice died with tumours, while 2 tumours developed in the treated group. The 6 treated mice died within 22 weeks, 2 of them with tumours.

*Experiment* 4 (0·025 mg. of methylcholanthrene, 75·0 mg. of testosterone propionate ; 10 control and 10 treated mice) :—During the 12th week of the experiment 5 mice suddenly died in the treated group. The first tumour appeared after 9 weeks in the control as compared with 16 weeks in the treated mice (Table II). After 14 weeks 3 control mice developed tumours, while none appeared in the treated mice. After 28 weeks 6 tumours occurred in the control mice and 3 tumours in the treated mice.

*Experiment* 8 (1·0 mg. of methylcholanthrene, 50·0 mg. of testosterone propionate ; 30 control and 30 treated mice, 15 of each sex) :—Injections of testosterone propionate were started 2 weeks before the administration of the carcinogen. All the mice were killed after 13 weeks and their tumours recorded. In cases where small nodules were present their nature was established by histological examination.

It is shown in Table IV that after 13 weeks 9 tumours were present in the 14 surviving control females (64 per cent), while an equal number of treated

*Testosterone Propionate on the Induction of Subcutaneous Tumours.*

deaths without tumours in weeks.
Denominator = mice dying without tumours.

| 23. | 24. | 25. | 26. | 27. | 28. | 29. | 30. | 31. | 32. | 33. | 34. | 35. | 36. | 37. | 38. | 39. | 40. | 41. | 42. | 43. | 44. | Suvivors without tumours after 44 weeks. | Effective total mice.§ | Total tumours. | Minimal latent time—weeks. | 50% latent time —weeks. |
|---|---|---|---|---|---|---|---|---|---|---|---|---|---|---|---|---|---|---|---|---|---|---|---|---|---|---|
| .. | .. | .. | .. | .. | .. | .. | .. | .. | .. | .. | .. | .. | .. | .. | .. | .. | .. | .. | .. | .. | .. | 0 | 10 | 10 | 10 | 10·8 |
| .. | .. | .. | .. | .. | .. | .. | .. | .. | .. | .. | .. | .. | .. | .. | .. | .. | .. | .. | .. | .. | .. | 0 | 10 | 10 | 15 | 15·4 |
| .. | .. | .. | .. | .. | .. | .. | .. | .. | .. | .. | .. | .. | .. | .. | .. | .. | .. | .. | .. | .. | .. | 2 | 19 | 15 | 9 | 11·6 |
| 1/0 | .. | .. | .. | .. | .. | .. | .. | 1/0 | .. | .. | 1/0 | .. | .. | .. | .. | .. | .. | .. | .. | 1/0 | .. | 3 | 19 | 13 | 13 | 15·7 |
| .. | .. | .. | .. | .. | .. | .. | .. | .. | .. | .. | .. | .. | .. | .. | .. | .. | .. | .. | .. | .. | .. | 0 | 7 | 5 | 8 | 9·4 |
| .. | .. | .. | .. | .. | .. | .. | .. | .. | .. | .. | .. | .. | .. | .. | .. | .. | .. | .. | .. | .. | .. | 0 | 5 | 2 | 16 | 16·0 |
| .. | 1/0 | .. | .. | .. | .. | 1/0 | .. | .. | .. | .. | .. | .. | 1/0 | 1/0 | .. | .. | .. | .. | 1/0 | .. | .. | 0 | 10 | 10 | 9 | 14·6 |
| .. | .. | .. | .. | .. | 1/0 | 1/0 | .. | .. | .. | .. | .. | .. | .. | .. | .. | .. | .. | .. | .. | .. | .. | 1 | 5 | 4 | 16 | 17·5 |
| 1/0 | 1/0 | .. | 1/0 | .. | .. | .. | .. | .. | .. | .. | .. | .. | .. | .. | .. | .. | .. | .. | 1/0 | .. | .. | 0 | 15 | 15 | 14 | 16·2 |
| 1/0 | 1/0 | .. | .. | .. | .. | .. | 0/1 | .. | .. | .. | 1/0 | .. | 0/1 | .. | .. | .. | .. | .. | .. | .. | .. | 0 | 13 | 10 | 17 | 17·6 |
| 1/0 | .. | .. | .. | .. | .. | .. | .. | 1/0 | .. | .. | .. | .. | .. | .. | .. | .. | 0/1 | .. | .. | .. | .. | 0 | 9 | 7 | 11 | 12·4 |
| .. | .. | .. | .. | .. | .. | .. | .. | 0/1 | .. | .. | .. | .. | .. | .. | .. | .. | .. | .. | .. | .. | .. | 2 | 8 | 4 | 14 | 15·0 |
| 2/0 | 1/0 | .. | .. | 1/0 | .. | .. | .. | .. | .. | .. | .. | 1/0 | .. | .. | .. | .. | .. | .. | .. | 0/3 | 0/1 | 7 | 20 | 9 | 14 | 15·2 |
| .. | .. | .. | 1/0 | .. | 1/0 | 0/2 | .. | .. | .. | .. | .. | .. | .. | 0/1 | 0/2 | .. | .. | .. | .. | .. | .. | 1 | 16 | 10 | 14 | 14·8 |

tr. = treated ; conc. = concentration.

permanently. † Removed after 8 weeks. ‡ Removed after 4

are excluded. ‖ Ten mice were implanted and 10 injected females developed only 3 tumours (21 per cent). The male mice did not show a clear-cut result. Five treated males died early without tumours. The surviving 10 males showed 4 tumours, while 15 control males developed 7 tumours. This difference is not statistically significant.

The total number of tumours in males and females was 16 out of 29 control mice (55·1 per cent) and 7 out of 24 treated mice (29·1 per cent)—a statistically significant difference.

B. *Mice implanted with methylcholanthrene-cholesterol pellets.*

*Experiment* 5 (1·0 mg. of methylcholanthrene, pellets left permanently; 75·0 mg. of testosterone propionate; 15 control and 15 treated mice):—The first tumour appeared after 14 weeks in the control and after 17 weeks in the treated mice. Three treated mice died without tumours between the 15th and 19th week (Table II). At the end of 16 weeks 5 tumours were present in the control and none in the treated group. After 32 weeks 14 control mice and 9 treated mice developed tumours. The average increase in weight of testosterone-treated mice during the first 8 weeks of treatment was the same as in the control mice. At the end of 15 weeks, however, a slight decrease in both control and treated mice occurred.

*Experiment* 6 (1·0 mg. of methylcholanthrene, pellets removed after 8 weeks; 40·0 mg. of testosterone propionate; 10 control and 10 treated mice):—The first tumour arose after 11 weeks in the control, and after 14 weeks in the treated mice (Table II). At the end of 13 weeks 3 tumours were found in the control and none in the treated group. Two control and 3 treated mice died early without tumours. After 36 weeks there were 7 tumours in the control and 4 tumours in the treated group.

*Experiment* 7 (1·0 mg. of methylcholanthrene, pellets removed after 4–5 weeks; 62·5–75·0 mg. of testosterone propionate; 20 control and 20 treated mice):—In both groups the first tumour developed after 14 weeks (Table II). At the end of 20 weeks 4 tumours were present in the control and 7 tumours in the treated mice. After 28 weeks 8 tumours occurred in the control, while 10 tumours were present in the treated group. This result differs from the results of all the other experiments (see p. 391).

The injection of testosterone propionate over a period of 10 to 12 weeks (Experiments 1, 2 and 4) or the implantation of approximately the same amount of the hormone (Experiments 2 and 3) delayed the appearance of subcutaneous tumours in mice receiving the carcinogen by about 4–5 weeks. No effect of testosterone propionate was observed in experiments in which the carcinogen was implanted in pellets and later removed.

The amount of hormone absorbed from the injected oily solution is not known. The amount absorbed from the implanted tablets was calculated from the initial weight of the tablets and the weight after removal on the death of the host. The rate of absorption was about 4 mg. per month. In mice surviving for more than 5 months the tablets were almost completely absorbed.

Sixty tumours were examined histologically. Fifty-six were sarcomata (93·3 per cent) and 4 skin carcinomata (6·7 per cent).

DISCUSSION.

In Experiment 1, in which 1·0 mg. of the carcinogenic agent was employed, all the control and treated mice died with tumours. This shows that testosterone had only a delaying influence, and was unable to prevent the carcinogenic action of methylcholanthrene.

In Experiments 2 to 4, in which smaller doses of methylcholanthrene were injected (0·25 mg. and 0·125 mg.), testosterone propionate caused not only delay in tumour appearance, but also a lower tumour incidence after 24 weeks. Twenty-six tumours developed in 36 control mice (72·2 per cent) and 14 tumours in 29 treated mice (48·2 per cent)—a statistically significant result.

In Experiments 5 to 7, 1·0 mg. of methylcholanthrene was implanted in 5 per cent hydrocarbon-cholesterol pellets. This method was at first considered to be more precise, because it was thought that the carcinogenic agent would be absorbed in uniform amounts over a given period. However, a comparison of the tumour incidence after injections and pellet implantations showed that the tumours did not occur earlier or at more regular intervals with the latter method.

In Experiment 6 the pellets were removed after 8 weeks, and in Experiment 7 after 4 to 5 weeks. In some mice the pellets were encapsulated in varying degrees, and the removal of the capsule in a number of mice could not be avoided. This is likely to have influenced the tumour incidence, and thus to have made a comparison between control and treated mice unreliable. Therefore, the results of Experiments 6 and 7 may be open to criticism. In these experiments no effect of testosterone propionate was observed. This was in contrast with the experiments in which methylcholanthrene was injected in olive oil. No adequate explanation can be given at present. However, the findings of the experiments on the induction of sarcomata in mice implanted with methylcholanthrene-cholesterol pellets by Shimkin and Wyman (1947) may be of interest. They implanted 0·002 to 0·5 mg. of methylcholanthrene in a 0·1 to 25 per cent concentration in cholesterol into $C_3H$ male mice. No shortening of the latent period was observed when higher concentrations and doses were implanted. The authors suppose that the localization of the hydrocarbon by means of cholesterol may have removed the differences in the latent period, as compared with that of the mice given the carcinogen in tricaprylin.

In the present experiments, in which 5 per cent hydrocarbon-cholesterol pellets were implanted and remained permanently under the skin, the latent period and the tumour incidence were similar to those in which mice were injected with the carcinogenic agent in much lower concentrations (0·4 to 0·1 per cent). However, when the pellets were removed after a short exposure (4 to 5 weeks) the latent period was longer and the incidence of tumours lower than in mice injected with lower concentrations of the carcinogen. This may have altered the results of testosterone treatment, because the bulk of the androgen was injected long before the tumours began to develop. The unequal removal of the carcinogenic agent in individual control and treated mice may also have been responsible for the negative result, because mice treated with testosterone were not discarded, even if it was felt that a small part of the carcinogenic pellet may have been left.

The need for larger groups of animals in experiments in which the influence on the latent period or on the incidence of tumours is investigated was fully realized. In the present experiments the large doses of testosterone propionate employed (about 8·0 g. of the hormone in all) precluded the use of larger groups of animals. Owing to the rather small number of mice employed in Experiments 1 to 7 the number of tumours induced in the control and experimental mice at 4-weekly intervals in each of the experiments was added and the tumour incidence compared (Table III). This was considered admissible, as the dose of the carcinogen was the same in both groups of each experiment and the dose of testosterone propionate was almost uniform (62·5 to 75·0 mg.).

TABLE III.—*Experiments* 1 *to* 7 : *Total Incidence of Tumours.*

| Weeks. | Control. Effective‡ total mice. | Control. Number of tumours. | Control. Per cent of tumours. | Treated. Effective‡ total mice. | Treated. Number of tumours. | Treated. Per cent of tumours. |
|---|---|---|---|---|---|---|
| 12 | 90 | 19 | 21·1 | 80 | 0 | 0 |
| 16 | 90 | 38 | 42·2* | 76 | 17 | 22·3* |
| 20 | 90 | 50 | 55·5† | 76 | 39 | 51·3† |

* This difference is statistically significant.
† This difference is not significant.
‡ Mice dying before the first tumour occurred are excluded.

The following results were obtained when the numbers of tumours in the control and treated mice occurring at 4-weekly intervals were compared. Up to the end of 12 weeks from the beginning of the experiments the results were clear-cut. There were no tumours in the 80 treated mice, and 19 tumours were observed in 88 control mice alive at that time. Between the 12th and 16th week the tumours began to appear in the treated mice. At the end of this period 38 tumours were found in the control (42·2 per cent) as compared with 17 tumours in the treated mice (22·3 per cent). Thus, although there were twice as many tumours in the control as in the experimental mice, approximately the same number of new tumours developed in both groups during these 4 weeks. Between the 16th and 20th week 12 new tumours appeared in the control and 22 in the treated mice. The total number of tumours reached 50 in the control and 39 in the treated mice.

A significant difference in the influence of testosterone propionate on the development of tumours in males and females was observed in Experiment 8 (Table IV).

TABLE IV.—*Experiment* 8 : *Incidence of Tumours after* 13 *Weeks.*

| Sex. | | Number of mice. | Effective† total mice. | Number of tumours and deaths without tumours.* | Per cent of tumours. |
|---|---|---|---|---|---|
| ♀ | Control | 15 | 14 | 9/1 | 64 |
| | Treated | 15 | 14 | 3/1 | 21 |
| ♂ | Control | 15 | 15 | 7/0 | 46 |
| | Treated | 15 | 10 | 4/5 | 40 |

* Numerator = mice with tumours. Denominator = mice dead without tumours.
† Mice dying before the first tumour occurred are excluded.

The massive doses of testosterone propionate employed did not cause any loss of weight in the treated mice. On the contrary, a slight increase in weight of the treated in comparison with the control mice was observed. Testosterone propionate exerted a marked influence on male and female genital organs :

hypertrophy of the seminal vesicles in the male, enlargement of the uterus and vagina, hypertrophy of the clitoris and an extreme hypertrophy of the clitoral glands in the female. Atrophy of the thymus and adrenals was the most striking finding in both sexes. Enlargement of the kidneys was also encountered. In addition, marked pigmentation of the mammary nipples in all dark-coated females (Flaks, 1948) and prolapse of the vagina and uterus in a number of mice was observed.

No connection could be found, at the present stage of the experiments, between the delaying influence of testosterone on carcinogenesis and the well-defined changes induced by the hormone in many organs. The hyperpigmentation of the nipples, which seems to be an outcome of increased activity of oxidation processes induced by testosterone propionate in the tissues, and indicated by the formation of black melanin, may prove to be of help in the understanding of the effect of testosterone on tumours.

The mechanism of the interference of testosterone with the action of methylcholanthrene is not known. Some workers consider the inhibitory influence of testosterone on spontaneous mammary tumours in mice as a castration effect. According to these workers, testosterone, by inhibiting the production of oestrogen in the ovaries inhibits the development of the mammary glands. They claim that this effect is similar to that first obtained by Lathrop and Loeb (1916), and Cori (1927), who showed that castration of female mice, at an early age, inhibited the development of breast cancer. The present experiments, in which an inhibitory influence on subcutaneous tumours induced by methylcholanthrene was only obtained after large doses of testosterone, suggest that this influence may not be hormonal. Doses which gave hormonal effects, such as masculinization, were not sufficient to produce even slight inhibition of tumours (Flaks and Ber, 1938*a*, 1938*b*). It is possible, therefore, that testosterone may directly interfere, at least temporarily, with the malignant transformation of the normal cell under the influence of methylcholanthrene.

### SUMMARY.

1. The influence of large doses of testosterone propionate (50·0–75·0 mg.) on the latent period and the incidence of subcutaneous tumours induced by 20-methylcholanthrene in 121 mice of several strains was investigated. One hundred and twenty-two mice served as controls.

2. An average delay of about 4 weeks in the appearance of 50 per cent of subcutaneous tumours induced by methylcholanthrene was observed after 62·5–75·0 mg. of testosterone propionate injected over a period of 10–12 weeks.

3. This result confirms and extends the findings of previous experiments in which the influence of testosterone propionate on skin cancer induced by painting with methylcholanthrene was studied.

4. The total incidence of tumours in mice treated with testosterone propionate was significantly reduced only up to the 16th week after the administration of the carcinogen (42·2 per cent to 22·3 per cent). No significant difference was observed later.

5. Implantation of testosterone propionate exerted a similar influence.

6. There was no decrease in weight of mice after large doses of testosterone propionate.

7. Testosterone propionate produced masculinization and hyperpigmentation of the mammary nipples in female mice. A few mice developed prolapse of the vagina and uterus.

8. The nature of the delaying influence of testosterone propionate on tumour induction remains to be elucidated.

9. Histological examination of 60 of the induced tumours revealed 56 sarcomata (93·3 per cent) and 4 squamous-celled carcinomata (6·7 per cent).

The author is indebted for the generous gift of "Testoviron" to Dr. R. L. Walsh and Mr. T. E. Riddle of British Schering Ltd., and to Dr. W. A. R. Thomson, of Boots Pure Drug Co., Ltd., for the testosterone propionate tablets.

### REFERENCES.

ANDERVONT, H. B., AND SHEAR, M. J.—(1942) *J. nat. Cancer Inst.*, **2**, 333.
CORI, C. F.—(1927) *J. exp. Med.*, **45**, 983.
FLAKS, J.—(1948) *J. Endocrinol.*, **5**, 259.
*Idem* AND BER, A.—(1938*a*) *C. R. Soc. Biol., Paris*, **128**, 506.—(1938*b*) *Bull. Ass. franç. Cancer*, **28**, 108.—(1939) *Proc. 3rd Int. Cancer Congr.*, p. 160.
HEIMAN, J.—(1944) *Cancer Res.*, **4**, 31.
JONES, E. E.—(1941) *Ibid.*, **1**, 787.
LACASSAGNE, A., AND RAYNAUD, A.—(1939) *C. R. Soc. Biol., Paris*, **131**, 586.
LATHROP, A. E. C., AND LOEB, L.—(1916) *J. Cancer Res.*, **1**, 1.
LOESER, A. A.—(1941) *Lancet*, ii, 698.
NATHANSON, I. T., AND ANDERVONT, H. B.—(1939) *Proc. Soc. exp. Biol., N.Y.*, **40**, 421.
SHEAR, M. J.—(1936) *Amer. J. Cancer*, **26**, 322.
SHIMKIN, M. B., AND WYMAN, R. S.—(1947) *J. nat. Cancer Inst.*, **8**, 49.